# Characterization of N‐Terminal Acetylated α‐Hemoglobin Stabilizing Protein (AHSP) by Top‐Down High‐Resolution Mass Spectrometry From Human Preterm Newborns Oral Fluid

**DOI:** 10.1002/rcm.10107

**Published:** 2025-07-17

**Authors:** Federica Iavarone, Chiara Tirone, Simona Fattore, Davide De Tomaso, Nicoletta Menzella, Giovanni Vento, Alessandra Olianas, Barbara Manconi, Tiziana Cabras, Giulia Guadalupi, Cristina Contini, Mozhgan Boroumand, Claudia Desiderio, Alexandra Muntiu, Antonella Fiorita, Matteo Fraschini, Vassilios Fanos, Gavino Faa, Irene Messana, Massimo Castagnola

**Affiliations:** ^1^ Dipartimento di Scienze Biotecnologiche di Base, Cliniche Intensivologiche e Perioperatorie Università Cattolica del Sacro Cuore Rome Italy; ^2^ Fondazione Policlinico Universitario “A. Gemelli”—IRCCS Rome Italy; ^3^ Neonatology Unit, Department of Woman and Child Health and Public Health Fondazione Policlinico Universitario “Agostino Gemelli,” IRCCS Rome Italy; ^4^ Dipartimento di Scienze Della Vita e Dell'ambiente, Sezione Biomedica Università di Cagliari Monserrato Italy; ^5^ National Institute on Aging, NIH Baltimore Maryland USA; ^6^ Istituto di Scienze e Tecnologie Chimiche “Giulio Natta,” Consiglio Nazionale Delle Ricerche Rome Italy; ^7^ Dipartimento di Neuroscienze, Organi di senso e Torace Fondazione Polcilinico Universitario “Agostino Gemelli,” Università Cattolica del Sacro Cuore, Rome, Italy Rome Italy; ^8^ Dipartimento di Ingegneria Elettrica ed Elettronica Università Degli Studi di Cagliari Cagliari Italy; ^9^ Neonatal Intensive Care Unit (NICU), Dipartimento di Scienze Chirurgiche Università di Cagliari Cagliari Italy; ^10^ Dipartimento di Scienze Mediche e Sanità Pubblica Università di Cagliari Monserrato Italy; ^11^ Department of Biology, College of Science and Technology Temple University Philadelphia Pennsylvania USA; ^12^ Unità di Proteomica Of the European Center for Brain Research‐ IRCCS Fondazione Santa Lucia Rome Italy

**Keywords:** AHSP, human preterm, LC–MS, proteomics

## Abstract

**Rationale:**

Alpha‐hemoglobin stabilizing protein (AHSP) is an erythroid‐specific protein forming a stable complex with free α‐hemoglobin, but not with β‐hemoglobin or hemoglobin A (α_2_β_2_), thus preventing harmful aggregation of α‐hemoglobin during normal erythroid cell development and avoiding its pro‐oxidant activity. Although its function has been extensively studied in erythroid cells, its presence in preterm newborns' oral fluid remains unexplored. Given the high susceptibility of preterm infants to hematological disorders, characterizing AHSP in their oral fluid could provide valuable insights into fetal erythropoiesis and its potential role as a biomarker for neonatal anemia and transfusion needs.

**Methods:**

The primary structure of AHSP was determined by high‐resolution top‐down proteomic analysis using a nano‐HPLC–ESI–MS/MS approach on oral fluid samples from preterm newborns. Specimens were collected non‐invasively from infants, promptly treated with formic acid, and analyzed with an Orbitrap‐based mass spectrometry platform. The intact protein's molecular mass and fragmentation pattern were assessed to determine its primary structure and post‐translational modifications. Statistical analysis was performed to explore correlations between AHSP presence and neonatal clinical parameters, including anemia and transfusion needs.

**Results:**

The experimental monoisotopic molecular mass value [M + H^+^]^1+^ at *m/z* 11744.958 ± 0.3 was inconsistent with the protein sequence reported in the literature, and the MS/MS fragmentation pattern was in agreement with the loss of the N‐terminal methionine residue followed by Nα‐terminal acetylation, a very common post‐translational modification, now recognized as having an important role in modulating protein function, localization and protein stability and turnover. The sporadic samples having detectable amounts of AHSP resulted from preterm newborns with severe anemic status, while all were submitted to iron supplementation.

**Conclusions:**

Although the data obtained so far cannot be used for quantitative analysis or statistical evaluation, AHSP appears to stand out as a potential early biomarker of neonatal hematological disorders, highlighting a new perspective for future investigations.

## 1 Introduction

Around the first years of 2000, our teams, at the Cagliari and the Catholic‐Policlinico Gemelli (Rome) Universities, started an investigation of the intact proteins of human saliva by an HPLC–MS top‐down pipeline [1]. Studies performed to investigate physiological variations of the human salivary proteome highlighted interesting age‐related modifications, especially in children up to puberty [2, 3, 4, 5]. In human preterm newborns, the oral fluid is commonly collected during their management to avoid its dangerous pouring inside the bronchus and the lungs, which allowed us to push our studies on the intact proteins of human saliva to the first days of life.

The first results, although obtained using less performing instruments, highlighted the profound differences that exist between the salivary proteome of children, adults, and preterm newborns [2, 6], prompting us to understand whether proteins absent in children and adults but present in newborns could have a potential role during fetal development. Studies performed with more advanced mass spectrometers allowed us to characterize numerous peptides and proteins, some investigated also during neonatal development by immunohistochemical methods using fetal autopsy tissues [6, 7].

In the present paper, we report on the characterization in the human preterm newborns' oral fluid of the alpha‐hemoglobin stabilizing protein (AHSP), which, to our knowledge, has never been described before in such biofluid. AHSP is an erythroid‐specific protein, forming a stable complex with free α‐hemoglobin, but not with β‐hemoglobin or hemoglobin A (HbA, α_2_β_2_), first identified by Kihm et al. using a screening for genes induced by the essential erythroid transcription factor GATA‐1 [8]. AHSP is specifically expressed in nucleated erythroid precursors during late‐stage erythropoiesis, primarily in bone marrow and fetal liver [5, 9], while it is not typically found in body fluids like plasma or saliva. For this reason, its appearance in oral fluid, particularly in preterm newborns, raises novel questions regarding AHSP's role in the perinatal context. To date, the known protein function is to serve as a chaperone for hemoglobin. In fact, AHSP selectively binds free alpha‐globin chains (in their heme‐bound form, α^0^Hb), stabilizing them and preventing their precipitation and oxidative toxicity [8, 10]. It does not interact with free beta‐globin or gamma‐globin chains and is absent in hemoglobin tetramers (HbA or HbF). Instead, AHSP functions as a transient chaperone, guiding α^0^Hb toward correct pairing with β‐globin to form adult hemoglobin (HbA, α_2_β_2_), or with γ‐globin to form fetal hemoglobin (HbF, α_2_γ_2_) [8]. Few studies also implicate AHSP in oxidative stress protection and red cell integrity [10, 11, 12], both of which are critical in the fragile neonatal and preterm erythropoietic environment. AHSP expression may be dysregulated or insufficient in preterm infants, potentially contributing to anemia of prematurity, a common condition marked by reduced hemoglobin levels and increased transfusion needs [13, 14]. Although no circulating or secreted forms of AHSP have been reported in adults, its detection in preterm oral fluid might suggest either altered erythroid dynamics or a possible local tissue source in perinatal tissues undergoing high turnover or stress. Overall, considering the high susceptibility of preterm infants to hematological disorders [13, 14], the characterization of AHSP in their oral fluid could provide valuable insights into fetal erythropoiesis, as well as its potential role as a biomarker for anemia of prematurity and transfusion needs. In this context, unlike the primary structure of AHSP reported in the Swiss Prot database (UniProt accession code Q9NZD4), high‐resolution MS/MS fragmentation experiments of the intact protein demonstrated that in oral fluids from preterm newborns, the protein loses the N‐terminal methionine residue (i‐Met) and undergoes subsequent Nα‐terminal acetylation.

## 2 Materials and Methods

### 2.1 Settings

The enrollment of newborns and the collection of saliva samples were performed at the *Unità Operativa Complessa di Neonatologia* of the *Fondazione Policlinico Universitario A. Gemelli IRCCS* of Rome. The treatment and proteomic analysis of the collected saliva samples were performed at the Neonatal Intensive Care Unit (NICU) of the *Dipartimento di Scienze Biotecnologiche di Base*, *Cliniche Intensivologiche e Perioperatorie* of the *Università Cattolica del Sacro Cuore* of Rome. The *Unità di Proteomica* of IRCCS‐*Fondazione Santa Lucia of Roma*, the *Dipartimento di Scienze della Vita e dell'Ambiente* and *Sezione di Anatomia Patologica* of the *Dipartimenti di Scienze Mediche e Sanità Pubblica*, and the Neonatal Intensive Care Unit (NICU) of the *Dipartimento di Scienze Chirurgiche* of Cagliari University and the *Istituto di Scienze e Tecnologie Chimiche* “Giulio Natta” of the *Consiglio Nazionale delle Ricerche* contributed to the analysis of saliva samples and to data processing.

### 2.2 Study Population and Inclusion Criteria

The study was carried out in accordance with the ethical standards laid down in the 1964 Declaration of Helsinki. All rules were observed, and written consent forms were signed by the parents of each newborn. For ethical reasons, the oral fluid was collected exclusively when sample collection caused no stress.

Twenty‐one preterm infants with gestational age between 175 and 216 days (25–30 weeks), admitted to the Neonatal Intensive Care Unit (NICU) were enrolled for this study. Oral fluid samples were collected weekly from the first week of life up to 40 weeks (286 days) of post conceptional age (PCA), or up to discharge if it occurred earlier. Infants with major congenital malformations or prenatal infections were excluded from the study. Globally, 161 specimens were serially collected from 21 preterm newborns. The study was approved by the ethics committee of the Catholic University (Prot.n. 0024661/21).

### 2.3 Chemicals

Formic acid (FA), water, and acetonitrile (ACN) LC–MS grade were from Merck (Darmstadt, Germany).

### 2.4 LC–MS Proteomic Analysis

The oral fluid was aspired from the mouth of preterm newborns by using a soft plastic pipette, immediately mixed in a 50% proportion with an aqueous FA solution (0.2%, *v/v*) and centrifuged at 14 000 *g* to remove the insoluble materials. Acidic supernatants were stored at −80°C until used. Oral fluid analyses have been carried out by UltiMate 3000 RSLC‐nano System coupled to Orbitrap Fusion Lumos Tribrid Mass Spectrometer and EASY‐Spray nanoESI (Thermo Fisher Scientific, Waltham, MA, USA) applying the chromatographic and mass spectrometer operating conditions below described. Chromatographic separation was performed on EASY‐Spray PepMap C18 column (15 cm in length × 50 μm of internal diameter [ID], 2‐μm particles, 100‐Å pore size) (Thermo Fisher Scientific) in coupling to Acclaim PepMap100 nano‐trap cartridge (C18, 5 μm, 100 Å, 300 μm i.d. × 5 mm) (Thermo Fisher Scientific). Separation was performed at 40°C in gradient elution, at mobile phase flow rate of 0.3 μL/min, using aqueous FA solution (0.1%, *v/v*) as Eluent A and ACN/FA solution (99.9:0.1, *v/v*) as Eluent B as follows: (i) 5% B (7 min), (ii) from 5% to 25% B (34.5 min), (iii) from 25% B to 55% (19.5 min), (iv) 90% B (2 min), (v) from 90% B (5 min), (vi) 90% B to 5% B (1 min), and (vii) 5% B (9 min). The injection volume was 5 μL. LC–MS operated in positive ionization mode at 120 000 full scan resolution (at *m/z* 400) in the *m/z* acquisition range 150–2000, performing MS/MS fragmentation by collision‐induced dissociation (CID, 35% normalized collision energy) and data‐dependent acquisition (DDA) scan mode. The minimum signal was set to 500.0, the isolation width at *m/z* 2, and the default charge state to +2. MS/MS spectra acquisition was performed in the Orbitrap mass analyzer. The MS/MS characterizations of AHSP have been deposited into the ProteomeXchange Consortium (http://www.ebi.ac.uk/pride) via the PRIDE [15] partner repository with the dataset identifier PXD061305. The mass spectrometer was routinely calibrated to ensure measurement accuracy, and the overall quality of the data was confirmed through the consistent detection of endogenous salivary proteins.

### 2.5 Data Acquisition

Thermo Xcalibur 2.2 computer program (Thermo Fisher Scientific) was used for data acquisition and interpretation with the aid of BLAST Expasy software (https://www.expasy.org/resources/uniprot‐blast) and Proteinprospector freeware tools (https://prospector.ucsf.edu/prospector/mshome.htm). Deconvolution was performed using the function Extract Raw file of the Xcalibur software (Thermo Fisher Scientific), which we use to elaborate our raw data, using the following parameters: 44% fit factor, 25% remainder threshold, minimum intensity set to 1, expected intensity error set to 3, and S/N threshold set to 2. The MS/MS spectra manual inspection data are in Data S1.

### 2.6 Quantification

The label‐free quantitation of AHSP was performed by measuring the area under the curve of the extracted ion current (XIC) peaks revealed by high‐resolution LC–MS analysis. The three most intense monoisotopic ions [M + 12H]^12+^ at *m/z* 980.29, [M + 11H]^11+^ at *m/z* 1069.32, and [M + 10H]^10+^ at *m/z* 1176.14, originating from the monoisotopic [M + H]^1+^ ion at *m/z* 11 744.958 of Nα‐acetylated AHSP missing the N‐terminal Met_1_, were used for this purpose. XIC peak area, expressed in arbitrary units, is proportional to protein concentration and under constant analytical conditions suitable for quantitative comparisons.

## 3 Results and Discussion

This study reports for the first time the determination of the primary structure of intact AHSP, establishing the N‐terminal loss of the initiator methionine residue (i‐Met) and Nα‐terminal acetylation by high‐resolution nano‐HPLC–ESI–MS investigation of the intact proteome present in the oral fluid from preterm newborns. Since its first detection [8], the structural characteristics of AHSP and its interaction with different hemoglobins have been the stimulus for many studies, as well reported in the outstanding review of Favero and Costa [12]. For this reason, accurate determination of its primary structure is pivotal for understanding the thermodynamics of these interactions.

### 3.1 Detection and Structural Characterization of AHSP

The high‐resolution HPLC–ESI–MS total‐ion current (TIC) elution profile of specimens serially collected from 21 newborns was investigated. N‐terminal acetylated AHSP was characterized in 19 specimens (11.8%), from 9 of the 21 newborns involved. Data regarding the enrolled infants are summarized in Table 1. The median gestational age was 29 weeks [27–30 weeks], and the median weight was 1080 g [800–1240 g].

**TABLE 1:** Relevant demographic and clinical data regarding the infants enrolled in the study.

*N*	21
Gestational age (weeks)	27.7 ± 2.3
Birth weight (grams)	978 ± 0.3
Male sex	12 (57%)
Vaginal delivery	6 (28%)
Prolonged rupture of membranes > 3 weeks	1 (4%)
Apgar 1′	6 [5–7]
Apgar 5′	8 [8–8.5]
Infants undergoing red blood cell transfusion	12 (57%)
Number of red blood cell transfusions	1 [0–6]
Iron supplementation	21 (100%)
Iron dose (mg/kg/die)	6 [5–6]

*Note:* Data are expressed as median [interquartile range], (percentage).

**FIGURE 1:**
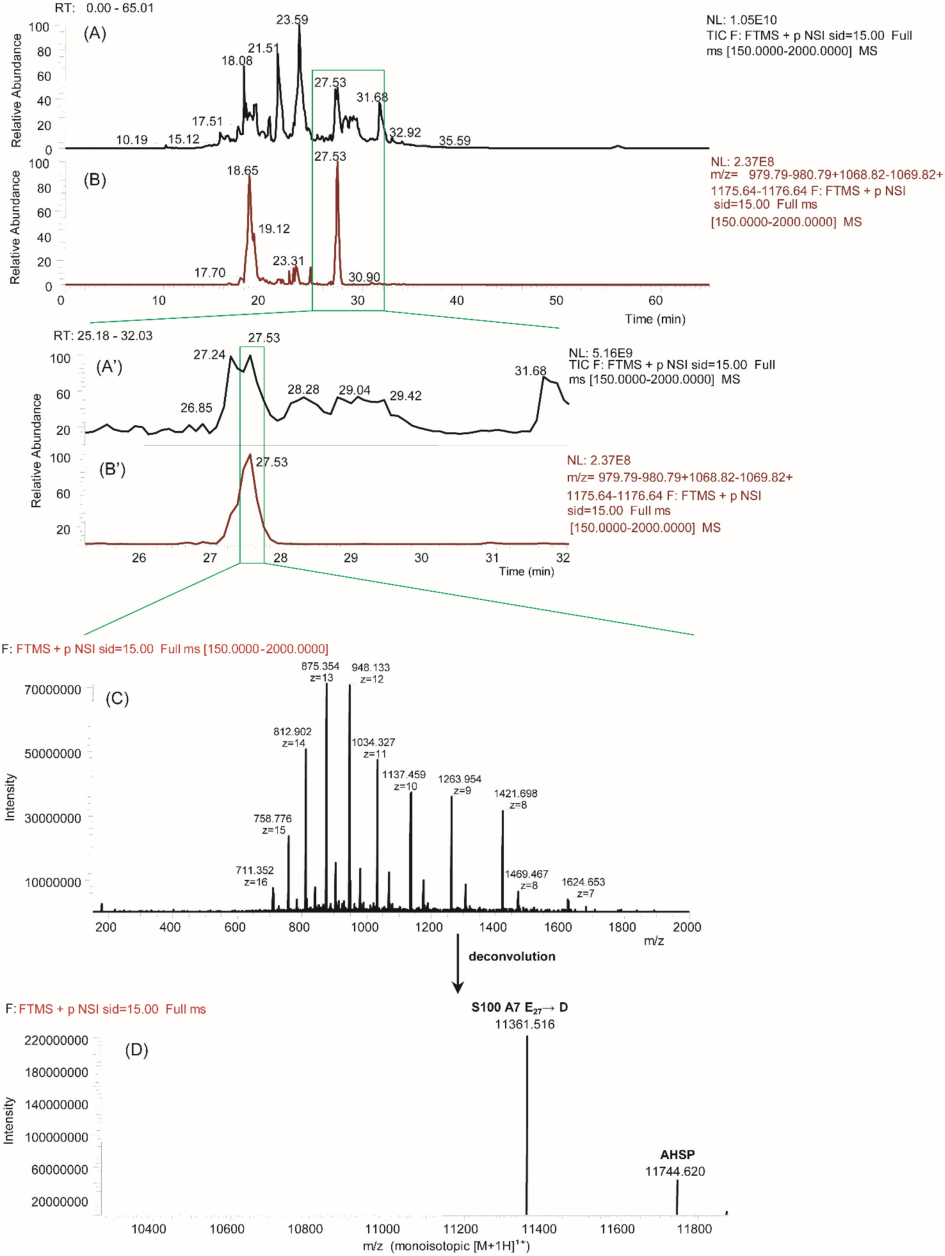
High‐resolution LC–MS detection of AHSP in the oral fluid of a preterm newborn positive for the protein. (A) Total‐ion current (TIC) profile of the oral fluid. (B) Extracted ion current (XIC) revealed by searching the three AHSP multicharged ions [M+12H]^12+^, [M+11H]^11+^, and [M+10H]^10+^ at *m/z* 980.29, 1069.32 and 1176.14, respectively. (A′, B′) Enlargements of Panels (A) and (B) between 25.18‐ and 32.03‐min interval of LC elution time. (C) ESI–MS spectrum recorded during the elution of the peak centered at 27.53 min. (D) Deconvolution of the ESI spectra shown in Panel (C). AV, averaged spectra; NL, normalization level; RT, retention time.

**FIGURE 2:**
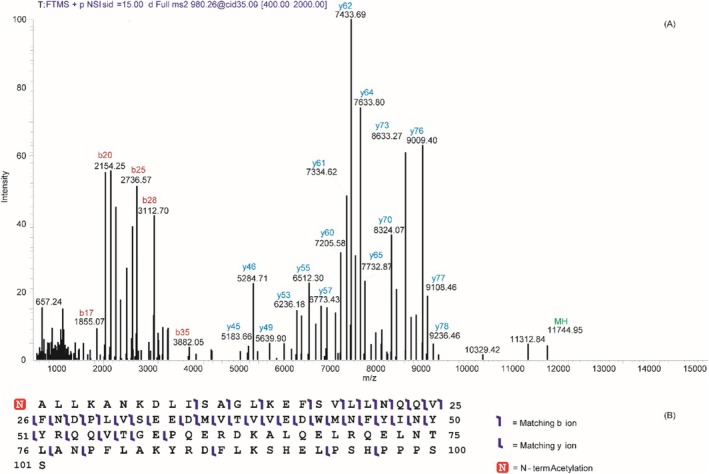
(A) MS/MS fragmentation CID spectrum of the multicharged ion [M+12H]^12+^ at *m/z* 980.29 of AHSP, selected and isolated in MS_1_ during a data‐dependent acquisition (DDA) at 27.53 min of LC elution time of the sample of Figure 1. (B) Observed MS/MS fragmentation of the sequence of AHSP as represented by ProSightLite. The spectrum is congruent to the ASHP sequence only assuming the loss of i‐Met_1_ followed by Nα‐terminal acetylation (in red). The accurate manual interpretation of the experimental MS/MS CID fragmentation (PRIDE dataset identifier PXD061305) is reported in Data S1.

The high‐resolution HPLC–ESI–MS TIC elution profile of the oral fluid of a human preterm newborn is reported in Figure 1A together with the enlargement between 25.18 and 32.03 min of elution time (Panels A′ and B′). The ESI–MS spectrum collected during the elution of the peak at 27.53 min, observable in Panel C, showed two main Gaussians suggesting the presence of at least two proteins. Indeed, deconvolution of the mass spectrum provided two main experimental monoisotopic [M + H]^1+^ values (Panel D), one at *m/z* 11361.516, attributed to the S100A8 E_27_ → D variant previously characterized with the loss of i‐Met and Nα‐terminal acetylation [2], and the other at *m/z* 11744.620, pertaining to an unknown protein. Figure 2 shows the deconvolution of the MS/MS spectrum obtained by CID fragmentation of the multicharged [M + 12H]^12+^ ion at *m/z* 980.29 (Panel A). The manual inspection of the **y**‐ion series allowed the identification of a short amino acid sequence trait that the BLAST tool identified as an internal region of AHSP. The theoretical fragmentation pattern of the AHSP obtained by the protein prospector tool for the relative UniProt accession code Q9NZD4 evidenced a shift of about −89 Da of the experimental ***b***‐ions, which extend from the amino terminus, while the experimental ***y***‐ions well fitted with the experimental data, suggesting the presence of an N‐terminal modification in the protein. Indeed, by supposing the elimination of the iMet_1_ and the acetylation of the Nα‐terminal Ala_2_, the experimental and theoretical fragmentation spectra, as well as the monoisotopic mass of the intact protein, were in optimal agreement (Data S1, Figure 2B).

Acetylation of the N‐terminal alanine residue was confirmed by the manual inspection of MS/MS spectra, which allowed the annotation of AHSP sequence. Specifically, the b5 ion and four internal fragments of the protein were diagnostic to locate acetylation at the N‐terminus, excluding the lysine residues at position 4 or 7 of the protein (Data S1). The internal fragments were the following: (1) position 5–13 (theoretical monoisotopic [M + H]^1+^ at *m/z* 870.47, experimental monoisotopic [M + H]^1+^ at *m/z* 870.47); (2) position 3–12 (theoretical monoisotopic [M + H]^1+^ at *m/z* 1054.63, experimental monoisotopic [M + H]^1+^ at *m/z* 1054.52); (3) position 3–21 (–H_2_O) (theoretical monoisotopic [M + H]^1+^ at *m/z* 2023.19, experimental monoisotopic [M + H]^1+^ at *m/z* 2023.16); (4) position 3–21 (–NH_3_) (theoretical monoisotopic [M + H]^1+^ at *m/z* 2024.18, experimental monoisotopic [M + H]^1+^ at *m/z* 2024.16.

### 3.2 Prevalence and Quantification of AHSP in Preterm Newborns

The presence of AHSP in the oral fluid of other preterm newborns was evaluated by searching in the TIC profiles the current pertaining to the three most intense monoisotopic ions [M + 12H]^12+^ at *m/z* 980.29, [M + 11H]^11+^ at *m/z* 1069.32, and [M + 10H]^10+^ at *m/z* 1176.14, which are generated from the monoisotopic [M + H]^1+^ ion at *m/z* 11744.958 of Nα‐acetylated AHSP missing the N‐terminal Met_1_ (Figure 1A′, B′). The area of the peak centered at 27.53 min shown in both Panels B and B′ of Figure 1 was utilized for label‐free relative quantification of AHSP and for the search of AHSP in 161 specimens serially collected from 21 preterm newborns.

In accordance with the guidelines and internal protocols, all infants received iron supplementation at different doses depending on their hematocrit levels, initially intravenously and subsequently, once enteral nutrition was tolerated, orally. Packed red blood cells (PRBCs) transfusions were administered in cases of severe anemia, following internal protocols. Overall, 12 out of 21 infants (57%) received red blood cell transfusions, with a median number of transfusions corresponding to 1 (reference number of transfusions from 0 to 6). AHSP was found only in 19 specimens (11.8%), from 9 newborns while it was not found at all in 12 newborns. The 9 newborns in whom AHSP was identified did not differ from the other 12 in baseline characteristics, such as gestational age and birth weight: the median gestational age was 26 weeks in the AHSP‐positive group [interquartile range 24.71–27.43] and 29.1 weeks in the group in which the protein was not identified [IQR 26.7–30.0], *p* value 0.363; birth weight was 1005 g in the first group [IQR 883–1088] and 1060 g in the second group [IQR 795–1203], *p* value 0.247 (Mann–Whitney *U* test).

In four newborns, AHSP was already present in the sample collected during the first week of life: in two of these cases, maternal blood loss/suspected placental abruption was reported. In the other two cases, the newborns were anemic at birth and received their first transfusion within the first week of life. In one of these infants, positivity was detected at 5, 9, and 12 weeks of life, but not in the intermediate samples. In the remaining five newborns, the first positive sample was detected later, at 3, 5, and 9 weeks of life, respectively. No significant correlation was found between age at the time of collection (in terms of days of life) and the positivity or level of AHSP. No temporal associations with PRBC transfusions were identified either. In the three pairs of monozygotic twins, concordance was observed: AHSP was detected in samples from one pair, while in the other two pairs, it was not. The newborns in whom the protein was identified had received a median of six transfusions [range 1–7]; in those in whom the protein was not identified, the median number was one transfusion [range 1–7] (Figure S1). The analysis of the data showed a positive correlation between the presence of AHSP in the infants' oral fluid samples and the number of transfusions required during hospitalization (Spearman's rho 0.186, p 0.018, Figure S1). No temporal association with transfusions was identified. Initial AHSP positivity was observed both in neonates who had not yet received any transfusions and in those who had previously undergone multiple transfusions but did not require further transfusion support thereafter. Using the paired Student's *T*‐test, we compared the AHSP, dividing the infants into two groups based on the need for transfusion therapy. The group that received transfusions showed significantly higher values (1.6 × 10^8^ ± 8.2 × 10^8^ vs. 1.0 × 10^6^ ± 5.0 × 10^6^, *p* = 0.024, data not shown).

### 3.3 AHSP Structural Interactions and Biological Implications

Various researches in the literature are devoted to the determination of the structural interaction of AHSP either with α‐hemoglobin, or with tetrameric adult hemoglobin (HbA, α_2_β_2_) or with fetal hemoglobin (HbF, α_2_γ_2_) [16]. Some of them were carried out evaluating the expression of the AHSP gene during erythropoiesis [16]. Because adequate amounts of the native AHSP are not available to carry out X‐rays [11], or circular dichroism (CD) or isothermal titration calorimetric experiments [9], all these studies utilized recombinant AHSP, including the N‐terminal methionine, as encoded by the DNA sequence, as well as an extra N‐terminal Gly‐Ser dipeptide added to generate a cleavage site recognized by thrombin. According to NMR results [17, 18], the secondary structure of the recombinant protein, 104 amino acid residues long, is arranged in a bundle of three elongated heterogeneous antiparallel α‐helices. AHSP exists in two forms, *cis* and *trans*, that are distinguished because of the arrangements of the loop region between helices 1 and 2 due to cis/trans isomerization of the Pro_30_ residue. However, once AHSP binds to αHb, Pro_30_ adopts just one conformation. The interaction with α‐hemoglobin is ensured by the contact of the loop situated between helix‐1 and helix‐2 domains, where Pro_30_ plays a pivotal role. The interaction of AHSP perturbs the proximal heme pocket of O_2_‐α‐hemoglobin throughout the modification of the orientation of the two distal (His_58_ [E7]) and proximal (His_87_ [F8]) histidine residues of α‐hemoglobin globin. The N‐terminal tail of AHSP is far away from the contact site, and the N‐terminal modification of the native protein here reported probably does not have a significant impact on this interaction. However, for the sake of accuracy, it should be considered that the N‐terminal acetylation here described replaces the three terminal amino‐acid residues of the recombinant isoform (Ala)‐Met‐Ser‐Gly‐NH_2_ and might have important roles in modulating protein localization, stability, and turnover. The N‐terminal sequence suggests that the enzymes involved in native AHSP post‐translational subsequent modifications are at first the ribosomal‐bound methionine aminopeptidases (MAP), responsible for i‐Met excision followed by the action of an Nα‐terminal acetyltransferase (NAT) probably pertaining to the Nat‐A family [19].

### 3.4 AHSP and Hemoglobin Assembly in Preterm Newborns

Notably, α‐hemoglobin, as well as A‐γ or G‐γ globin (or both), was detected in almost all the samples positive for the presence of AHSP, while β‐hemoglobin was rarely detected, principally in those from newborns with PCA close to that of full‐term newborns. In this regard, the detection of AHSP in preterm newborn oral fluid, although sporadic, suggests a facilitative role in the assembly of α^0^‐globin chain with Aγ^0^‐globin chain or with Gγ^0^‐globin chain to generate a full cooperative HbF (α_2_γ_2_) during fetal development. This aligns with the experiments of Pinho et al. [20], which utilized rt‐hairpin RNA expression vectors aimed at the AHSP mRNA inhibition into K562 and CD34+ cells. K562 and CD34+ cells were stimulated to erythroid differentiation and examined in terms of gene expression, reactive oxygen species (ROS) production, apoptosis, and Hb synthesis [21]. RNA interference‐mediated knockdown of AHSP expression resulted in considerable α^0^‐globin precipitation, as well as in a significant decrease in HbF formation, increased ROS production, and increased rate of apoptosis.

## 4 Conclusions

This study provides the first detailed characterization of the primary structure of native AHSP in preterm newborn oral fluid, confirming the N‐terminal loss of the initiator methionine (i‐Met) and the presence of Nα‐terminal acetylation through high‐resolution nano‐HPLC–ESI–MS analysis. Our results suggest a positive correlation between the presence of AHSP in the preterm oral fluid specimens and the number of transfusions required during hospitalization, with a corresponding significantly higher value of AHSP in the group of infants that received transfusions. Although all the newborns studied were undergoing iron supplementation, our findings agree with those of Camila dos Santo et al. [21]. These authors found that AHSP in vitro binds and detoxifies free hemoglobin (Hb) in the body. This is crucial in conditions where an excess of free Hb is present, for instance, in the presence of iron overload due to red blood cell transfusions. Our data suggest that the sporadic detection of AHSP in the specimens is probably linked with a more severe state of anemia requiring red blood cell transfusion. Further studies are needed to assess the levels of AHSP in the oral fluid of preterm and at‐term neonates, correlating them with the timing of red blood cell transfusions.

## Author Contributions

**Chiara Tirone:** writing – review and editing, resources, visualization, conceptualization. **Simona Fattore:** writing – review and editing, resources. **Davide De Tomaso:** writing – review and editing, resources. **Nicoletta Menzella:** writing – review and editing, resources. **Giovanni Vento:** writing – review and editing, resources, conceptualization. **Alessandra Olianas:** writing – review and editing, formal analysis. **Barbara Manconi:** writing – review and editing, visualization, formal analysis. **Tiziana Cabras:** writing – review and editing, writing – original draft, formal analysis. **Giulia Guadalupi:** writing – review and editing, visualization. **Cristina Contini:** writing – review and editing, visualization, writing – original draft, formal analysis. **Mozhgan Boroumand:** writing – review and editing, writing – original draft, formal analysis. **Claudia Desiderio:** writing – review and editing, investigation, conceptualization, formal analysis. **Alexandra Muntiu:** writing – review and editing, investigation, formal analysis. **Antonella Fiorita:** writing – review and editing, conceptualization, writing – original draft. **Matteo Fraschini:** writing – review and editing, formal analysis. **Vassilios Fanos:** writing – review and editing, conceptualization, writing – original draft. **Gavino Faa:** writing – review and editing, conceptualization, writing – original draft, formal analysis. **Irene Messana:** writing – review and editing, writing – original draft, conceptualization, methodology, formal analysis. **Massimo Castagnola:** conceptualization, writing – original draft, writing – review and editing, methodology, formal analysis.

## Conflicts of Interest

The authors declare no conflicts of interest. All authors have read and agreed to the published version of the manuscript.

## Supporting information


Data S1.

**Figure S1.** Correlation between number of packed red blood cell (PRBCs) transfusions performed during hospitalization (on the y‐axis) and the presence (=1) or absence (=0) of AHSP in the newborn’s saliva sample (on the x‐axis).

## Data Availability

The mass spectrometry proteomics data have been deposited to the ProteomeXchange Consortium via the PRIDE partner repository with the dataset identifier PXD061305.

## References

[1] I. Messana , F. Loffredo , R. Inzitari , et al., “The Coupling of RP‐HPLC and ESI‐MS in the Study of Small Peptides and Proteins Secreted In Vitro by Human Salivary Glands That Are Soluble in Acidic Solution,” Eur J Morphol. 41, no. 2 (2003): 103–106, 10.1080/09243860412331282228. 15621864

[2] M. Castagnola , R. Inzitari , C. Fanali , et al., “The Surprising Composition of the Salivary Proteome of Preterm Human Newborn,” Molecular and Cellular Proteomics 10, no. 1 (2011): M110.003467, 10.1074/mcp.M110.003467. PMC3013458 20943598

[3] T. Cabras , E. Pisano , R. Boi , et al., “Age Dependent Modifications of the Human Salivary Secretory Protein Complex,” Journal of Proteome Research 8, no. 8 (2009): 4126–4134, 10.1021/pr900212u. 19591489

[4] B. Manconi , T. Cabras , E. Pisano , et al., “Modifications of the Acidic Soluble Salivary Proteome in Human Children From Birth to the Age of 48 Months Investigated by a Top‐Down HPLC‐ESI‐MS Platform,” Journal of Proteomics 91 (2013): 536–543, 10.1016/j.jprot.2013.08.009. 23973467

[5] I. Messana , T. Cabras , F. Iavarone , et al., “Chrono‐Proteomics of Human Saliva: Variations of the Salivary Proteome During Human Development,” Journal of Proteome Research 14, no. 4 (2015): 1666–1677, 10.1021/pr501270x. 25761918

[6] M. Arba , F. Iavarone , F. Vincenzoni , et al., “Proteomic Characterization of the Acid‐Insoluble Fraction of Whole Saliva From Preterm Human Newborns,” J Proteomics. 146 (2016): 48–57, 10.1016/j.jprot.2016.06.021. 27321913

[7] T. Cabras , B. Manconi , A. Olianas , et al., “The Characterization of Preterm Newborn Saliva by Top‐Down Proteomic as a Stimulus for the Study of Human Development. A Review of the Results Obtained Over the Past 25 Years,” Journal of Pediatric and Neonatal Individualized Medicine 14, no. 1 (2025): e140108, 10.7363/140108.

[8] A. J. Kihm , Y. Kong , W. Hong , et al., “An Abundant Erythroid Protein That Stabilizes Free α‐Haemoglobin,” Nature 417, no. 6890 (2002): 758–763, 10.1038/nature00803. 12066189

[9] D. Gell , Y. Kong , S. A. Eaton , M. J. Weiss , and J. P. Mackay , “Biophysical Characterization of the α‐Globin Binding Protein α‐Hemoglobin Stabilizing Protein,” Journal of Biological Chemistry 277, no. 43 (2002): 40602–40609, 10.1074/jbc.M206084200. 12192002

[10] X. Yu , Y. Kong , L. C. Dore , et al., “An Erythroid Chaperone That Facilitates Folding of Alpha‐Globin Subunits for Hemoglobin Synthesis,” J Clin Invest. 117, no. 7 (2007): 1856–1865, 10.1172/JCI31664. 17607360 PMC1904324

[11] L. Feng , D. A. Gell , S. Zhou , et al., “Molecular Mechanism of AHSP‐Mediated Stabilization of α‐Hemoglobin,” Cell 119, no. 5 (2004): 629–640, 10.1016/j.cell.2004.11.025. 15550245

[12] M. E. Favero and F. F. Costa , “Alpha‐Hemoglobin‐Stabilizing Protein: An Erythroid Molecular Chaperone,” Biochemistry Research International 2011 (2011): 373859, 10.1155/2011/373859. 21490703 PMC3070166

[13] L. F. Holzapfel , M. A. Rysavy , and E. F. Bell , “Red Blood Cell Transfusion Thresholds for Anemia of Prematurity,” NeoReviews 24, no. 6 (2023): e370–e376, 10.1542/neo.24-6-e370. 37258497 PMC10865726

[14] J. A. Widness , “Pathophysiology of Anemia During the Neonatal Period, Including Anemia of Prematurity,” NeoReviews 9, no. 11 (2008): e520, 10.1542/neo.9-11-e520. 20463861 PMC2867612

[15] Y. Perez‐Riverol , J. Bai , C. Bandla , et al., “The PRIDE Database Resources in 2022: A Hub for Mass Spectrometry‐Based Proteomics Evidences,” Nucleic Acids res. 50, no. D1 (2022): D543–D552, 10.1093/nar/gkab1038. 34723319 PMC8728295

[16] C. O. dos Santos , A. S. Duarte , S. T. Olalla Saad , and F. F. Costa , “Expression of Α‐Hemoglobin Stabilizing Protein Gene During Human Erythropoiesis,” Experimental Hematology 32, no. 2 (2004): 157–162, 10.1016/j.exphem.2003.11.002. 15102476

[17] C. M. Santiveri , J. M. Pérez‐Cañadillas , M. K. Vadivelu , et al., “NMR Structure of the α‐Hemoglobin Stabilizing Protein: Insights Into Conformational Heterogeneity and Binding,” Journal of Biological Chemistry 279, no. 33 (2004): 34963–34970, 10.1074/jbc.M405016200. 15178680

[18] C. F. Dickson , A. M. Rich , W. M. D'Avigdor , et al., “Α‐Hemoglobin‐Stabilizing Protein (AHSP) Perturbs the Proximal Heme Pocket of Oxy‐α‐Hemoglobin and Weakens the Iron–Oxygen Bond,” Journal of Biological Chemistry 288, no. 27 (2013): 19986–20001, 10.1074/jbc.M112.437509. 23696640 PMC3707698

[19] S. Calis and K. Gevaert , “The Role of Nα‐Terminal Acetylation in Protein Conformation,” FEBS Journal 292, no. 3 (2025): 453–467, 10.1111/febs.17209. 38923676

[20] F. O. Pinho , D. M. de Albuquerque , S. T. Olalla Saad , and F. F. Costa , “Reduction of AHSP Synthesis in Hemin‐Induced K562 Cells and EPO‐Induced CD34+ Cells Leads to α‐Globin Precipitation, Impairment of Normal Hemoglobin Production, and Increased Cell Death,” Experimental Hematology 36, no. 3 (2008): 265–272, 10.1016/j.exphem.2007.11.003. 18179859

[21] C. O. dos Santos , L. C. Dore , E. Valentine , et al., “An Iron Responsive Element‐Like Stem‐Loop Regulates α‐Hemoglobin‐Stabilizing Protein mRNA,” Journal of Biological Chemistry 283, no. 40 (2008): 26956–26964, 10.1074/jbc.M802421200. 18676996 PMC2555993

