# Supplementary material for: Characterization of N‐Terminal Acetylated α‐Hemoglobin Stabilizing Protein (AHSP) by Top‐Down High‐Resolution Mass Spectrometry From Human Preterm Newborns Oral Fluid
Source: Rapid Commun Mass Spectrom. 2025 Jul 17;39(21):e10107. doi: 10.1002/rcm.10107 (PMC12272030; doi:10.1002/rcm.10107)

**Supplementary Figure S1**

Correlation between number of packed red blood cell (PRBCs) transfusions performed during hospitalization (on the y-axis) and the presence (=1) or absence (=0) of AHSP in the newborn's saliva sample (on the x-axis).


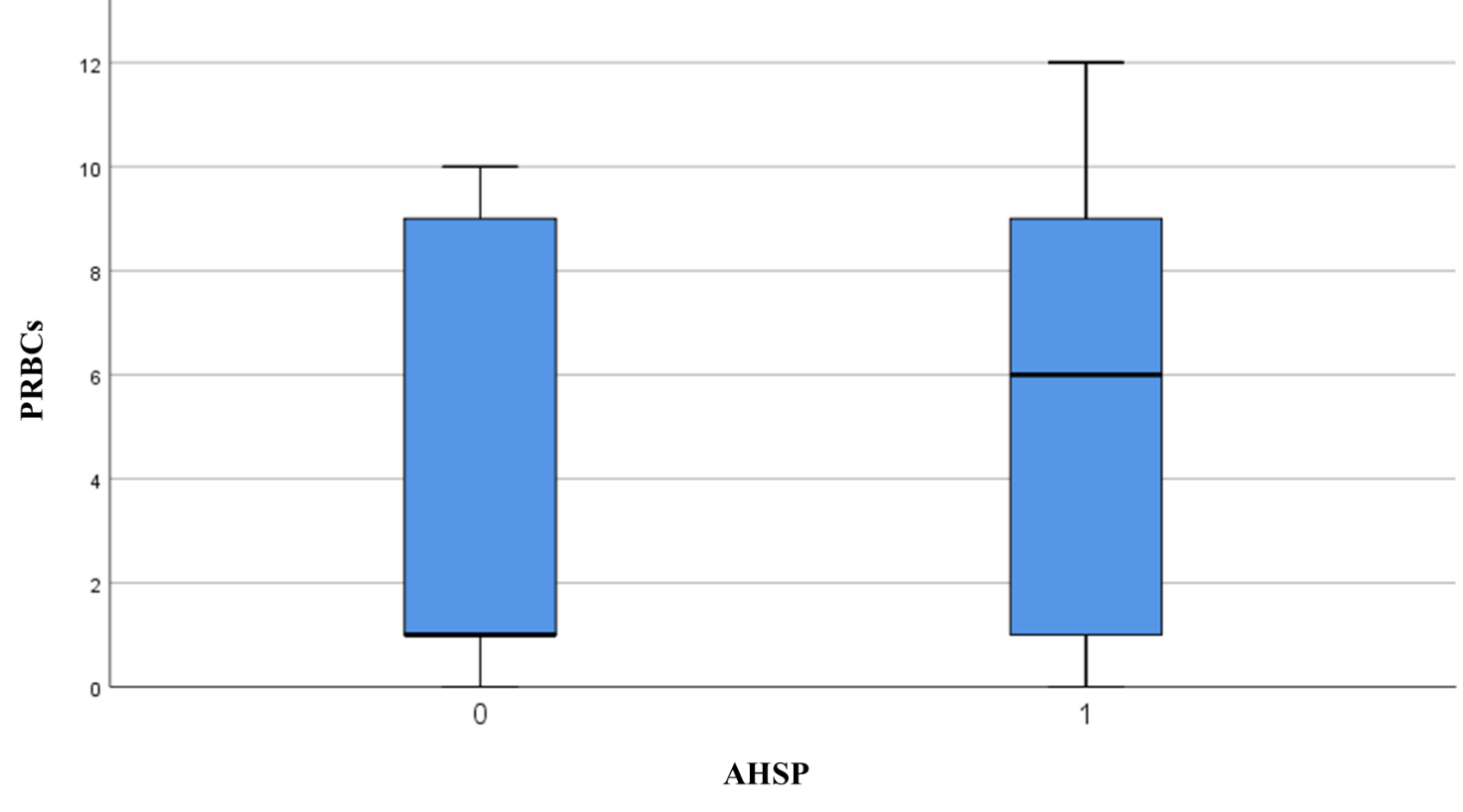

Supplement: Supplementary file 2 — Figure S1. Correlation between number of packed red blood cell (PRBCs) transfusions performed during hospitalization (on the y‐axis) and the presence (=1) or absence (=0) of AHSP in the newborn’s saliva sample (on the x‐axis). [file RCM-39-e10107-s002.docx]
